# Dissection of the signal transduction machinery responsible for the lysyl oxidase-like 4-mediated increase in invasive motility in triple-negative breast cancer cells: mechanistic insight into the integrin-β1-NF-κB-MMP9 axis

**DOI:** 10.3389/fonc.2024.1371307

**Published:** 2024-05-28

**Authors:** Fan Jiang, Youyi Chen, Nahoko Tomonobu, Rie Kinoshita, Ni Luh Gede Yoni Komalasari, Carlos Ichiro Kasano-Camones, Kazumi Ninomiya, Hitoshi Murata, Ken-ichi Yamamoto, Yuma Gohara, Toshiki Ochi, I Made Winarsa Ruma, I Wayan Sumardika, Jin Zhou, Tomoko Honjo, Yoshihiko Sakaguchi, Akira Yamauchi, Futoshi Kuribayashi, Junichiro Futami, Eisaku Kondo, Yusuke Inoue, Shinichi Toyooka, Masakiyo Sakaguchi

**Affiliations:** ^1^ Department of Cell Biology, Okayama University Graduate School of Medicine, Dentistry and Pharmaceutical Sciences, Okayama, Japan; ^2^ Department of Breast Surgery, The First Affiliated Hospital, Zhejiang University School of Medicine, Hangzhou, China; ^3^ Faculty of Medicine, Udayana University, Denpasar, Bali, Indonesia; ^4^ Faculty of Science and Technology, Division of Molecular Science, Gunma University, Kiryu, Gunma, Japan; ^5^ Department of Neurology, Okayama University Graduate School of Medicine, Dentistry and Pharmaceutical Sciences, Okayama, Japan; ^6^ Medical Oncology Department of Gastrointestinal Tumors, Liaoning Cancer Hospital & Institute, Cancer Hospital of the Dalian University of Technology, Shenyang, Liaoning, China; ^7^ Department of Interdisciplinary Science and Engineering in Health Systems, Okayama University, Okayama, Japan; ^8^ Department of Microbiology, Tokushima Bunri University, Sagamihara, Tokushima, Japan; ^9^ Department of Biochemistry, Kawasaki Medical School, Kurashiki, Okayama, Japan; ^10^ Division of Tumor Pathology, Near InfraRed Photo-Immuno-Therapy Research Institute, Kansai Medical University, Osaka, Japan; ^11^ Department of General Thoracic Surgery and Breast and Endocrinological Surgery, Okayama University Graduate School of Medicine, Dentistry and Pharmaceutical Sciences, Okayama, Japan

**Keywords:** breast cancer, invasion, lysyl oxidase, NF-κB, MMP9

## Abstract

**Background:**

Triple-negative breast cancer (TNBC) cells are a highly formidable cancer to treat. Nonetheless, by continued investigation into the molecular biology underlying the complex regulation of TNBC cell activity, vulnerabilities can be exposed as potential therapeutic targets at the molecular level. We previously revealed that lysyl oxidase-like 4 (LOXL4) promotes the invasiveness of TNBC cells via cell surface annexin A2 as a novel binding substrate of LOXL4, which promotes the abundant localization of integrin-β1 at the cancer plasma membrane. However, it has yet to be uncovered how the LOXL4-mediated abundance of integrin-β1 hastens the invasive outgrowth of TNBC cells at the molecular level.

**Methods:**

LOXL4-overexpressing stable clones were established from MDA-MB-231 cells and subjected to molecular analyses, real-time qPCR and zymography to clarify their invasiveness, signal transduction, and matrix metalloprotease (MMP) activity, respectively.

**Results:**

Our results show that LOXL4 potently promotes the induction of matrix metalloprotease 9 (MMP9) via activation of nuclear factor-κB (NF-κB). Our molecular analysis revealed that TNF receptor-associated factor 4 (TRAF4) and TGF-β activated kinase 1 (TAK1) were required for the activation of NF-κB through Iκβ kinase kinase (IKKα/β) phosphorylation.

**Conclusion:**

Our results demonstrate that the newly identified LOXL4-mediated axis, integrin-β1-TRAF4-TAK1-IKKα/β-Iκβα-NF-κB-MMP9, is crucial for TNBC cell invasiveness.

## 1 Introduction

Cancer cells have multiple strategies for surviving in the living body, such as exploiting angiogenesis as a nutrition supplier, utilizing metastasis to secure optimal sites for growth, and using immune tolerance in order to evade anti-cancer immunity. In all these cases, the cancer cells actively leverage their interstitial conditions (1, 2). Therefore, a deeper understanding of the complex machinery of the cancer stroma would be highly useful for developing treatments to prevent or ameliorate cancer progression (3–5). Accumulating evidence indicates that lysyl oxidase (LOX) family members play vital roles in the metastatic outgrowth of multiple cancer cell types (6) via not only an enzymatic collagen cross-linking that leads to rearrangement of extracellular matrices of the cancer stroma but also by manipulating the cancer cell surface (although the cell surface protein(s) targeted in such manipulation remain unknown). The former of these mechanistic strategies confers metastatic advantages to cancer cells by promoting their preferred stroma conditions, and the latter strategy confers metastatic advantages by accelerating proliferation and invasiveness (7, 8).

In our recent studies, some progress has been made in these areas. We have compiled a growing body of evidence that lysyl oxidase-like 4 (LOXL4) is a more advanced promoter of the cancer invasive outgrowth of breast cancer cells, and particularly that of triple-negative-breast-cancer (TNBC) cells, than the other LOX family proteins (LOX, LOXL1, LOXL2, and LOXL3) (9). We confirmed that LOXL4 can lead to collagen cross-linking modification coupled with dense network formation of new cancer blood vessels, thereby playing plays a general enzymatic role across multiple interactions, as seen in all of the LOX family members. On the other hand, our efforts have led to the identification of a cell surface client protein of LOXL4, annexin A2, for its-mediated cross-linking in TNBC cells. The LOXL4-mediated cross-linking yields polymerized annexin A2, which is required for membrane anchoring, resulting in significant accumulation of integrin-β1 at the membrane. Finally, the highly accumulated integrin-β1 at the cell surface functions to upregulate the attachment, proliferation, and invasion of TNBC cells (10). Although the enhanced binding of integrin-β1 to its substrate and following heightened growth cycling are attributed to integrin-β1, the mechanism underlying the accelerated invasiveness of TNBC cells remains elusive. In the present study, therefore, we sought to elucidate this mechanism at the molecular level.

We here show for the first time that the LOXL4-mediated cell surface accumulation of annexin A2/integrin-β1 potentiates TNBC cells to give rise to invasion at a significant level by promoting the transcriptional activity of the matrix metalloprotease 9 (MMP9) gene that potently upregulates its secreted protein in the mature active form. Our molecular-based analysis unravels the main signal cascade beneath the integrin-β1 to induce MMP9—that is, the TNF receptor-associated factor 4 (TRAF4)-TGF-β activated kinase 1 (TAK1)-nuclear factor-κB (NF-κB) axis. Our newly identified mechanism highlights the vital role of LOXL4 in the invasiveness of TNBC cells and provides evidence that could be used to develop an LOXL4-signal cascade targeted therapy as an effective treatment for metastatic TNBC.

## 2 Materials and methods

### 2.1 Cells and reagents

A human embryonic kidney cell line stably expressing the SV40 large T antigen: HEK293T (RIKEN BioResource Center, Tsukuba, Japan), a human luminal breast cancer cell line: MCF-7 cells (ATCC, Rockville, MD), human TNBC cell lines: MDA-MB-231 (ATCC), MDA-MB-436 (ATCC), BT-549 (ATCC), and HCC3153, which was kindly provided by Dr. Adi F. Gazdar (Hamon Center for Therapeutic Oncology Research and Department of Pathology, the University of Texas Southwestern Medical Center at Dallas, Dallas, TX, USA) were all cultivated in DMEM/F-12 medium (Thermo Fisher Scientific, Waltham, MA) supplemented with 10% FBS (Thermo Fisher Scientific). NF-κB activation inhibitor III (SM-7368) was purchased from Cayman Chemical (Ann Arbor, MI). MMP-9-IN-1 (another name: OUN87710, Selleck Chemicals, Houston, TX) and MG-132 (Selleck Chemicals) were used to block the MMP9 catalytic activity and suppress cell proteasome activity, respectively. BAPN, an irreversible lysyl oxidase inhibitor, was purchased from Merck Sigma-Aldrich (St. Louis, MO). In the cell cultures, these inhibitors were used at the final concentration of 10 µM for NF-κB activation inhibitor III, 10 µM for MMP-9-IN-1, 10 µM for MG-132, and 500 µM for BAPN, respectively.

### 2.2 Plasmids

The mammalian gene expression constructs used in this study were all made using the pIDT-SMART-C-TSC vector (pCMViR-TSC) as the backbone to express the cargo genes at significantly high levels (11). The cDNAs located on the multi-cloning site of the pCMViR-TSC were designed to be expressed in a C-terminal 3Myc-6His-tagged or 3HA-6His-tagged form. The cDNAs encoding GFP, the wild-type (wt) of LOX family members (LOX, LOXL1, LOXL2, LOXL3, and LOXL4), the wt of ITGB1, dominant negative TRAF4 (TRAF4 dn, N-terminal 266 aa deletion), and catalytic unfunctional kinase-dead TAK1 (TAK1 KD; the lysine at amino acid position 63 from the N-terminal start methionine is replaced by methionine) were inserted into the multi-cloning site of the pCMViR-TSC. Transient transfection of these plasmids into cultured cells was performed using FuGENE-HD (Promega BioSciences, San Luis Obispo, CA). To obtain stable transformants, we used our original vector named pSAKA-4B. A couple of cDNAs encoding GFP and the wt of LOX family members were inserted into the multi-cloning site of pSAKA-4B. The MDA-MB-231 cell clones stably overexpressing these genes were established by a convenient electroporation gene delivery method and subsequent selection with puromycin at 20 µg/mL. The establishment of MDA-MB-231 cell clones stably overexpressing LOXL4 mutCA (MDA-MB-231 LOXL4 mutCA) and the LOXL4 gene-ablated MDA-MB-231 cell clones (MDA-MB-231 knock-out (KO)) was reported previously.

### 2.3 siRNA

Human integrin-β1 (ITGB1 gene) siRNA (siITGB1, ID: s7575) and control siRNA (silencer select negative control No. 1 siRNA) purchased from Thermo Fisher Scientific were used for gene down-regulation experiments as previously reported (10).

### 2.4 Invasion assay

The invasion assay was performed using the Boyden chamber method as previously described. In brief, 8-μm pore filters set in Transwell culture inserts (Corning, Corning, NY) were coated with Matrigel. Cells were placed in the upper chamber in a low-serum medium, DMEM/F-12 (0.5% FBS), and the lower chamber was filled with the high-serum version (10% FBS) of the same medium. After 24 hours, cells that had passed through the membrane were stained with hematoxylin-eosin (H&E) (Muto Pure Chemicals, Tokyo). Each Transwell insert was microscopically imaged in five distinct regions at 10× in triplicate. The number of cells migrated to the five different areas was counted using BZ-analysis application software (Keyence) and summed as the total cell number.

### 2.5 Wound scratch assay

The migratory ability of cancer cells was determined through a wound-healing assay. Cells (6×10^4^ cells) were seeded into each well of the 24-well-collagen-coated plate and cultured to be 90-100% confluence in cell density. Then, a wound was made in the central region of the cell monolayer by scratching the plate with the edge of a 200 μL-pipette wide tip. After rinsing the scratched cell monolayer with phosphate-buffered saline (PBS) twice, each well was filled with DMEM/F-12 medium with 0.5% FBS. Images of the migration area were captured using an optical microscope at the zeroth or 12th hour (12).

### 2.6 Pull-down and immunoprecipitation

An IP assay of the expressed foreign proteins was performed using anti-HA tag antibody-conjugated agarose beads (Merck Sigma-Aldrich) or anti-DYKDDDDK tag antibody-conjugated agarose beads (WAKO, Osaka, Japan) that bind FLAG tag sequence. The pull-down and IP experiments were repeated at least three times. As previously reported, the IP of endogenous proteins was also investigated using an avidin-biotin interaction. Rabbit anti-human integrin-β1 antibody (Proteintech, Rosemont, IL) was biotinylated using a Biotin Labeling Kit-SH (Dojindo Molecular Technologies, Kumamoto, Japan) to recover antibody-free samples after IP using streptavidin-agarose (Thermo Fisher Scientific). All agarose beads used were preincubated with an albumin-based blocking buffer (5% bovine albumin, 6% glycine, 0.1% Tween-20 in PBS) before incubating with the cell extracts.

### 2.7 Western blotting

WB analysis was performed under conventional conditions. In brief, cell lysates were prepared using M-PER cell lysis buffer (Thermo Fisher Scientific). They were then supplemented with SDS-sample buffer and subjected to electrophoresis on SDS polyacrylamide gel electrophoresis (SDS-PAGE) gel. The proteins separated according to their molecular masses were then transferred onto a polyvinylidene difluoride (PVDF) membrane (Thermo Fisher Scientific) using a semi-dry blotter (Nihoneido, Tokyo). The membrane was incubated with a skim milk-based blocking buffer (10% bovine skim milk, 6% glycine, 0.1% Tween-20 in PBS) and then exposed to primary antibodies except for the anti-albumin antibody. Because bovine skim milk has a large amount of albumin that prevents the binding of anti-albumin to the blotted albumin, the blocking buffer was used a Casein-based blocking buffer (10% Casein, 6% glycine, 0.1% Tween-20 in PBS). The WB was repeated three times for each set of samples. The antibodies used were as follows: mouse anti-HA tag antibody (clone 6E2; Cell Signaling Technology, Danvers, MA), mouse anti-Myc tag antibody (clone 9B11; Cell Signaling Technology), rabbit anti-human MMP9 antibody (Cell Signaling Technology), rabbit anti-human Akt antibody (Cell Signaling Technology), rabbit anti-human phospho (T308)-Akt antibody (Cell Signaling Technology), rabbit anti-human phospho (S473)-Akt antibody (Cell Signaling Technology), rabbit anti-human NF-κB antibody (Cell Signaling Technology), mouse anti-human phospho (S536)-NF-κB antibody (Cell Signaling Technology), rabbit anti-human IKKα antibody (Cell Signaling Technology), rabbit anti-human IKKβ antibody (Cell Signaling Technology), rabbit anti-human phospho (S176/180)-IKKα/β antibody (Cell Signaling Technology), mouse anti-human Iκβα antibody (Cell Signaling Technology), rabbit anti-human phospho (S32)-Iκβα antibody (Cell Signaling Technology), rabbit anti-human integrin-β1 antibody (Proteintech), rabbit anti-human TRAF4 antibody (Proteintech), rabbit anti-human TAK1 antibody (Cell Signaling Technology), rabbit anti-DsRed (RFP) (Takara Bio USA, Mountain View, CA), rabbit anti-GFP (Thermo Fisher Scientific), mouse anti-β-actin (Merk Sigma-Aldrich), and rabbit anti-human albumin (Agilent’s DAKO, Glostrup, Denmark) that cross-reacts with bovine albumin with high affinity in WB.

### 2.8 Gelatin zymography

Zymography was used to detect the activity of MMPs in the cell-conditioned media. The 10-fold condensed cell-conditioned media were mixed with non-reducing SDS-sample buffer with no dithiothreitol (DTT) and applied to SDS-PAGE using 8% polyacrylamide gel, and then the electrophoresis was run. After electrophoresis, SDS was removed via incubation in 2% Triton X-100 at 37°C for 30 min. The gel was then transferred to 0.05 M Tris-HCl buffer (pH 8.0) containing five mM CaCl_2_ and incubated at 37°C for 18 h in the presence or absence of a specific inhibitor of MMP9 (MMP-9-IN-1, used at the final concentration of 10 µM), and stained with 1% Coomassie brilliant blue R-250 (FUJIFILM Wako Pure Chemical, Osaka, Japan). The gelatinolytic activity was detected as clear bands against a blue background of undegraded substrate.

### 2.9 Fluorescence-activated cell sorter-based cell sorting

To collect selectively the RFP-expressing cells in the transiently transfected cells, the post-transfected cell pellet was resuspended in 2 ml of FACS buffer and run through a 70 µm filter. The RFP-positive signal was measured using the BD FACSAria III (BD Biosciences, San Jose, CA), and data were analyzed using FlowJo software (BD Biosciences). Cell debris, doublets, and dead cells were gated out. The RFP-positive signal was detected and discerned by comparing it to that in non-transfected control cells. After broadly sorting cells according to the strength of their RFP signals (low to high), the positively sorted cells were collected from the cartridge. The collected cells (1 x 10^5^ cells) were seeded and used for the subsequent experiments.

### 2.10 Electrophoretic mobility shift assay

Nuclear extracts from cells were prepared according to the manufacturer’s instructions using NE-PER Nuclear and Cytoplasmic Extraction Reagents (Thermo Fisher Scientific), and EMSA for NF-κB was performed using a LightShift Chemiluminescent EMSA kit (Thermo Fisher Scientific) as previously reported. The 5’-biotin-labeled double-stranded NF-κB probe (forward: 5’-agttgaGGGGACTTTCCcaggc-3’; reverse: 3’-gcctgGGAAAGTCCCCtcaact-5’) was purchased from Merck Sigma-Aldrich. After the reactions, all samples were fractionated by 7% PAGE and blotted onto a Biodyne B nylon membrane (Thermo Fisher Scientific).

### 2.11 Statistical analysis

Each experiment was repeated three times, and the resulting raw data were statistically analyzed. The calculated values are the mean ± standard deviation (SD). We used a simple pair-wise comparison with Student’s *t*-test. Values of p<0.05 were considered statistically significant.

## 3 Results

### 3.1 Elevated invasive motility in LOXL4-overexpressing cells is associated with MMP9

**Figure 1:**
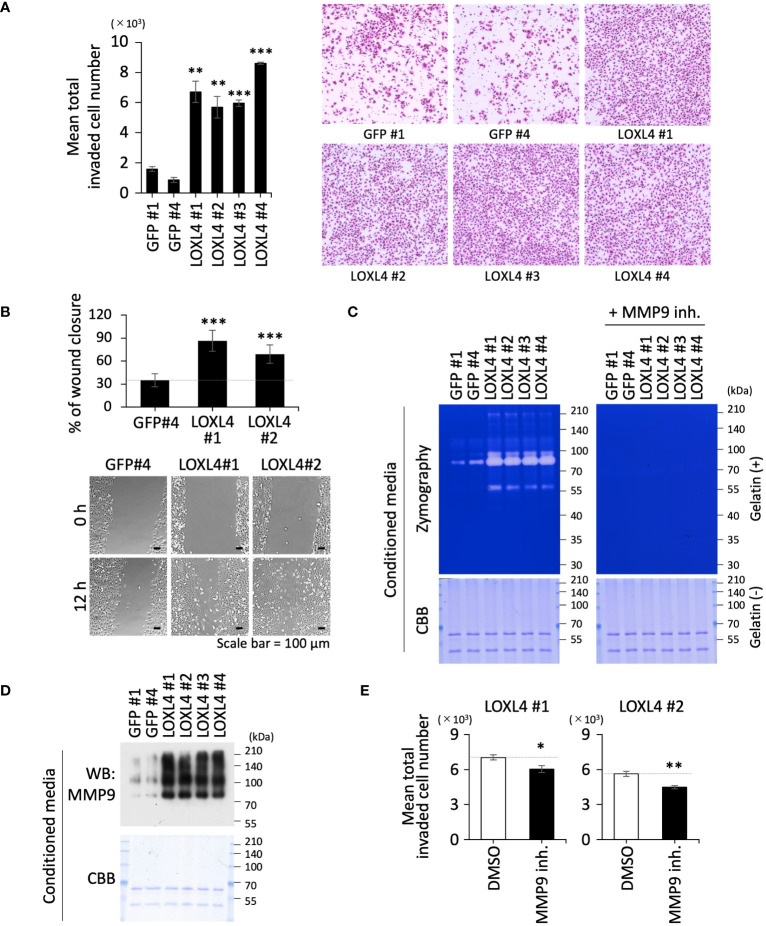
The vital role of LOXL4 in the invasion via induction of MMP9 in triple-negative breast cancer (TNBC) cells. **(A)** A trans-chamber-based cell invasion assay was performed for the indicated gene-engineered cells. The quantified data are displayed on the left side, and representative staining images are shown on the right. **(B)** A wound healing scratch assay was performed for the indicated gene-engineered cells. The quantified data are displayed on the upper side, and representative cell distribution images are shown on the lower side. **(C)** Zymography using gelatin substrate was performed to detect secretory MMP activity from the indicated cells. To specify the MMP9-mediated digestive bands, the electrophoresed gels were subjected to the reaction buffer with or without an MMP9-specific inhibitor. The loaded samples were checked for concurrent contents by visualizing the included proteins in another CBB-stained standard gel with no gelatin. **(D)** The same samples used in the zymography **(C)** were also applied to detect MMP9 protein by WB analysis. **(E)** Invasion assay set with or without MMP9 inhibitor was carried out to evaluate the effect of MMP9 on the LOXL4-enhanced invasiveness. Data are mean ± SD. *p<0.05, **p<0.01, ***p<0.001.

We previously revealed that LOXL4 is significantly heightened at both mRNA and protein levels in TNBC cells in the *in vitro* culture condition, which can give rise to significant levels of cellular invasion in TNBC cells (10). As a first step in our present investigation, we confirmed that the increase in LOXL4 expression at the TNBC cell culture levels is indeed reflected in the clinical breast cancer patient tissue specimens with an aggressive grade (**Supplementary Figure 1A**), implying that the elevated LOXL4 empowers cancer cells to possess a fierce invasive motility in the patients. We therefore tried to replicate previous results about the LOXL4-mediated invasive outgrowth in newly established gene-engineered cell clones that overexpress the wild-type (wt) LOXL4 (MDA-MB-231/LOXL4 wt) sustainably (**Supplementary Figure 1B**). As in our previous report, all the clones exhibited a very high capacity for invasiveness (**Figure 1A**) and migration (**Figure 1B**). Mining our previous RNA-Seq data for ZEB1 regulatable genes gave us a piece of important information—namely, the expressions of several MMP genes are followed by changes in the levels of LOXL4 expression (9). Owing to the importance of MMPs in cancer-invasive events, we next performed a screening for MMP(s) whose levels correlate with LOXL4-overexpression and thus could be used in the induction of LOXL4 expression. Real-time-quantitative PCR analysis using primers’ collection through all the MMP family (**Supplementary Figure 1C**) yielded two candidates, MMP1 and MMP9, which were considerably elevated in the LOXL4 clone compared to their levels in the control GFP clone (**Supplementary Figure 1D**). To examine whether the induction events of MMP1 and MMP9 are enzymatically functional in the extracellular space, gelatin substrate-based zymographic analysis was conducted for the condensed cell-conditioned culture media. The gelatin is a suitable substrate for MMP1 and MMP9. By this approach, we were able to determine that MMP9 is more readily induced than MMP1 since the digesting activity of gelatin, as manifested by the negative-stained bands, all disappeared into almost nothing when we treated the PAGE gel with a specific inhibitor of MMP9 for all duration long of the enzymatic reaction, and thus MMP9 acted as a more enzymatically mature protein in all the LOXL4 clones (**Figure 1C**). We found that these increased levels of the MMP9 enzymatic activities were attributed to the highly upregulated MMP9 at protein levels in the LOXL4 clones (**Figure 1D**). We then confirmed that the MMP9 inhibitor lessened the elevation of LOXL4-mediated invasion, confirming that MMP9 contributed to promoting cellular invasiveness conferred by LOXL4 (**Figure 1E**).

### 3.2 The crucial role of NF-κB in the transcriptional induction of MMP9 beneath the integrin-β1

The event of MMP9 induction led us to ask how LOXL4 enhances the promotion of MMP9 expression. One clue may come from the fact that the NF-κB transcription factor strongly induces MMP9 transcription in several types of cancer cells (13, 14). In addition, in our previous work, we have shown that the LOXL4 secreted from TNBC cells enzymatically acts to cross-link the cell surface annexin A2 in addition to cross-linking collagen, resulting in multimerized annexin A2 on the cell surface, which leads to an abundance of cell surface annexin A2. The abundant annexin A2 is effectively fashioned into the annexin A2/integrin-β1 complex, whereby integrin-β1 is enriched on the cell surface, contributing to cancerous behaviors such as enhanced growth and invasion (10). Interestingly, there have been reports suggesting that NF-κB is activated upon activation of the integrin-β1 (15, 16). It is thus conceivable that NF-κB is a crucial factor in the induction of MMP9, which is leveraged by the LOXL4-mediated abundance of integrin-β1 on the cell surface.

**Figure 2:**
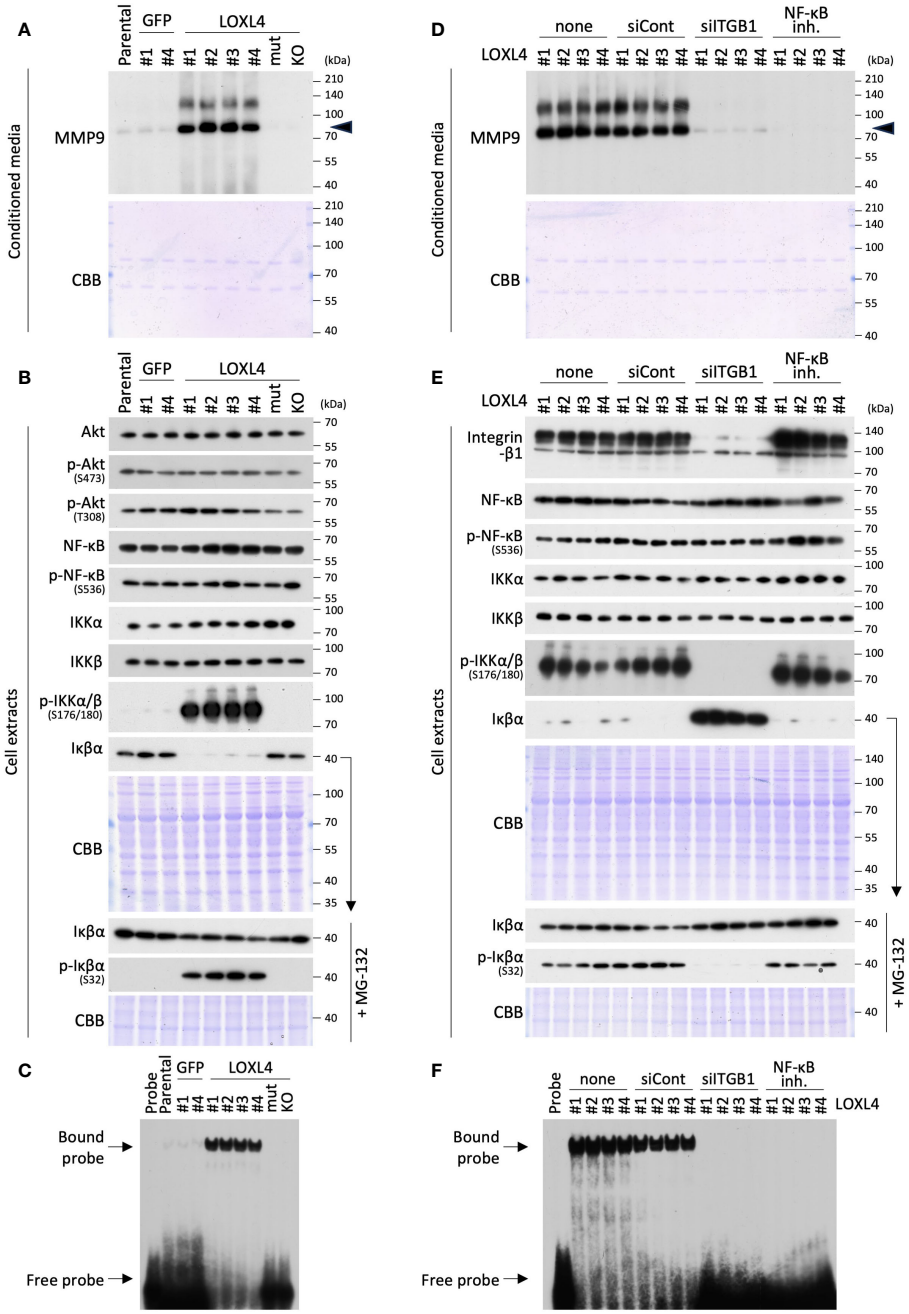
LOXL4-mediated regulation of the MMP9 through the activation of NF-κB. **(A)** The prepared conditioned media from one-day serum-free cultivation of the indicated cells were condensed 10-fold. The media specimens were subjected to SDS-PAGE and then applied to WB analysis to detect MMP or CBB staining as a sample control of proper loading. **(B)** The prepared whole cell extracts from the indicated cells under the standard culture were also subjected to SDS-PAGE and analyzed by WB for the proteins of interest. CBB visualized the loaded proteins and displayed the stained image as a control of proper sample loading. On the other hand, to correctly detect the phosphorylation levels of Iκβα, the indicated cells were all treated with MG-132 proteasome inhibitor to restore the Iκβα protein levels from their lowered levels caused by proteasome-mediated degradation. **(C)** The prepared nuclear extracts from the indicated cells were subjected to EMSA analysis to detect NF-κB activity. **(D, E)** WB analysis was done for the 10-fold condensed cell conditioned media and cell extracts from the LOXL4 wt-overexpressing clones to detect MMP9 **(D)** and indicated protein molecules **(E)** in a similar manner as described for panels **(A)** and **(B)**, except for the treatment with siRNAs (control siRNA: siCont; ITGB1 siRNA: siITGB1) or NF-κB inhibitor. **(F)** EMSA analysis of NF-κB was also performed for the nuclear extracts from the same series of cells, as shown in **(E)**.

To investigate this possibility, we conducted a signal analysis to determine whether NF-κB is a central factor in the tethering between MMP9 and integrin-β1 in the LOXL4-overexpressed TNBC cells (**Figures 2A–C**). Much like the zymographic analysis (**Figure 1C**), the signal analysis showed that the MMP9 band was constantly detected at a significant level in the condensed cell-conditioned culture media from the MDA-MB-231/LOXL4 wt clones (**Figure 2A**). On the other hand, no bands appeared in the media from the previously established gene-engineered cell clones overexpressing either the catalytically mutant-type (mutCA) of LOXL4 (MDA-MB-231/LOXL4 mutCA) or the genetically ablate intrinsic LOXL4 gene produced by CRISPR/Cas9-mediated gene editing (MDA-MB-231/LOXL4 KO) (10). We next examined vital proteins that have been reported to induce NF-κB activation upon integrin-β1 activation in cancer cells using the same series of cells shown in **Figure 2A**. Although activation-linked phospho-Akt and -NF-κB exhibited no apparent fluctuation in protein band levels, a highly notable induction of phospho-IκB kinase kinase (IKKα/β) was observed in LOXL4 wt clones but not control GFP or LOXL4-mutCA and -KO clones (**Figure 2B**). Concomitant with the increase in phosphorylation modification of IKKα/β, Iκβα was significantly lowered to an almost undetectable level. To confirm that the decrease in Iκβα was due to proteasome-mediated degradation coupled with the phosphorylation modification of Iκβα in the LOXL4 wt clones, we discretely treated each of the studied cell types with the proteasome inhibitor MG-132. By this approach, we found that the Iκβα bands in the LOXL4 wt clones were restored to levels similar to those in GFP and LOXL4-mutCA and -KO cells, and the enhanced phosphorylation was selectively increased in the Iκβα bands in the LOXL4 wt clones. Considering these results for the crucial signal dynamics, we then examined the NF-κB activation levels for the same cell types. The electromobility shift assay (EMSA) revealed that NF-κB activation was markedly elevated in the LOXL4 wt clones (**Figure 2C**). In addition, by the orthogonal experimental approach using a promoter reporter assay we confirmed that LOXL4 activates both the NF-κB responsive element (**Supplementary Figure 1E**) and the MMP9 promoter (**Supplementary Figure 1F**). These results indicate that the catalytic activation of LOXL4 is linked to NF-κB activation, and the latter activation is caused by the IKKα/β-mediated Iκβα phosphorylation following its proteasomal degradation.

To consolidate the results, we further studied the contribution of integrin-β1 to the detected pathway and the significance of NF-κB activation on the induction of MMP9 in the LOXL4 wt-overexpressing clones (**Figures 2D–F**). Interestingly, the siRNA-mediated-RNA interference using the siITGB1 validated previously and in this study (**Figure 2E**) and the chemical (NF-κB activation inhibitor III)-mediated inhibition of NF-κB activation both effectively inhibited MMP9 induction (**Figure 2D**) without any appreciable cytotoxicity under the experimental settings used (data not shown). In the case of the NF-κB activation inhibitor III, we confirmed that this inhibitor strongly suppressed the activation of NF-κB (**Figure 2F**) without any effect on the phosphorylation levels of IKKα/β or the phosphorylation or proteasomal degradation of Iκβα (**Figure 2E**). Our subsequent analysis of the signal proteins identified by western blotting (WB) (**Figure 2E**) and EMSA (**Figure 2F**) revealed the significance of integrin-β1 in the IKKα/β activation-Iκβα degradation-NF-κB activation pathway that is leveraged by LOXL4, since siITGB1 strongly blocks phosphorylation of IKKα/β, and adversely affects the increase in Iκβα protein, whose phosphorylation levels were markedly reduced. Our findings thus underscore the importance of the integrin-β1-IKKα/β-Iκβα-NF-κB axis for the LOXL4-mediated MMP9 induction.

### 3.3 Identification of upstream factors that activate the IKKα/β-Iκβα-NF-κB axis

**Figure 3:**
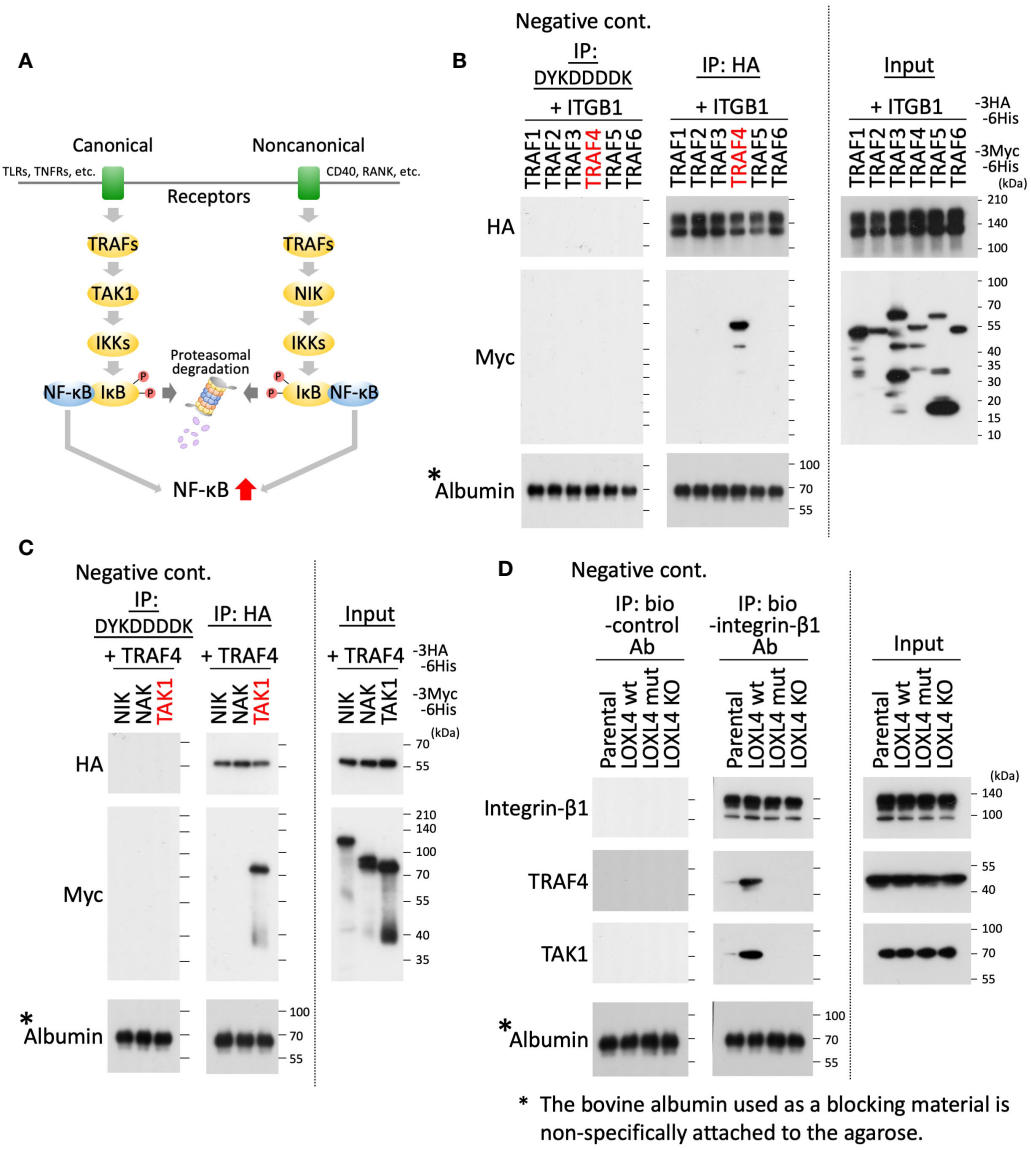
Identifying TRAF4 and TAK1 on the integrin-β1 signal cascade that links to NF-κB activation. **(A)** Schematic representation of the NF-κB activation flow via canonical and noncanonical pathways. **(B, C)** As indicated, the prepared cell lysates of HEK293T transfectants with different combinations of the plasmids were divided into three aliquots. The first was used as the whole cell lysate, i.e., as input (rightmost). The second was used to conduct immunoprecipitations with anti-HA tag antibody-conjugated agarose beads (middle). The remaining third was used to perform similar immunoprecipitations with anti-DYKDDDDK tag antibody-conjugated agarose beads as a negative control for the immunoprecipitation experiment (leftmost). After immunoprecipitation, the expressed products with beads-bound foreign proteins were analyzed by WB with an anti-HA, anti-Myc, or anti-albumin antibody. **(D)** The indicated cell extracts were incubated with the biotinylated (bio)-integrin-β1 antibody to capture the intrinsic integrin-β1 of the individual cells. On the other hand, bio-control IgG was used as a negative control for the same immunoprecipitation experiment. The captured integrin-β1 was collected by the pull-down method with avidin beads, and the precipitates were further subjected to WB with the indicated antibodies.

Several studies have investigated the presence of canonical and noncanonical signal pathways that lead to NFκB activation, which are signaled from membrane proteins (**Figure 3A**). To continue this line of investigation, we investigated which TRAF(s) is recruited to the integrin-β1. Using an interaction screening approach, we newly identified TRAF4 as the integrin-β1 interactive TRAF (**Figure 3B**). We then further investigated which kinases among TGF-β-activated kinase 1 (TAK1), NF-κB-inducing kinase (NIK), and NF-κB-activating kinase (NAK) interact with TRAF4. NAK was also used as a spare candidate to expect the binding to TRAF4. The effort revealed that TAK1 bound to the TRAF4 in the overexpression system (**Figure 3C**). The intrinsic interaction for the identified molecules was finally confirmed in the indicated cells (MDA-MB-231 parental cells, MDA-MB-231/LOXL4 wt, mt, and KO cells); that is, TRAF4 and TAK1 were both detected as defined bands in the integrin-β1 interaction experiment using the LOXL4 wt-overexpressing cells, and also as weak-to-slight bands in the parental cell extract (**Figure 3D**). Finally, we found that this interaction was diminished in both the LOXL4 mut-overexpressing cells and LOXL4-deleted KO cells.

### 3.4 The avid involvement of TRAF4 and TAK1 in the integrin-β1-IKKα/β-Iκβα-NF-κB-MMP9 axis

To gather additional evidence regarding the significance of the newly revealed involvement of TRAF4 and subsequent TAK1 molecules in the LOXL4-mediated integrin-β1-IKKα/β-Iκβα-NF-κB-MMP9 axis in TNBC cells, we conducted experiments similar to that shown in **Figures 2D–F**, except for the method of cell collection after the plasmid transfection. In this experiment, we used a dominant negative TRAF4 (TRAF4 dn) or kinase-dead TAK1 (TAK1 KD) expression plasmid to block each function in the plasmid-delivered cells according to a standard temporal transfection method. The blocking efficiency with foreign products is dependent on transfection efficiency. Still, unfortunately, the entry rate of the plasmid into cells was only around 40% for MDA-MB-231 cells, so we decided to draw and collect the plasmid-entry cells selectively using the fluorescence-activated cell sorting (FACS) apparatus. We have successfully employed this approach in several previous transfection experiments. In the transient transfection experiment using two different plasmids with two different cargo genes, respectively, one foreign gene-expressed cell showed positive expression for the other ectopic gene at a very high odds ratio of over 90%. Taking the actual double transfection result for GFP- with TRAF4 dn- or TAK1 KD-expression plasmids in this study as an example, the overlap rates of TRAF4 dn/GFP and TAK1 KD/GFP were 93% and 91%, respectively (**Supplementary Figure 2A**). According to the typical rule of plasmid entry into cells, we conducted experiments for the collected cells by cell sorting for the RFP-positive cells and their conditioned-condensed media. Because LOXL4 wt-overexpressing stable clones are all already GFP-positive by the used plasmid trait, we used an RFP-expressing plasmid as the cell sorting marker instead of the GFP-plasmid for the transient double transfection combined with TRAF4 dn, TAK1 KD, or control empty plasmid vector (EV).

**Figure 4:**
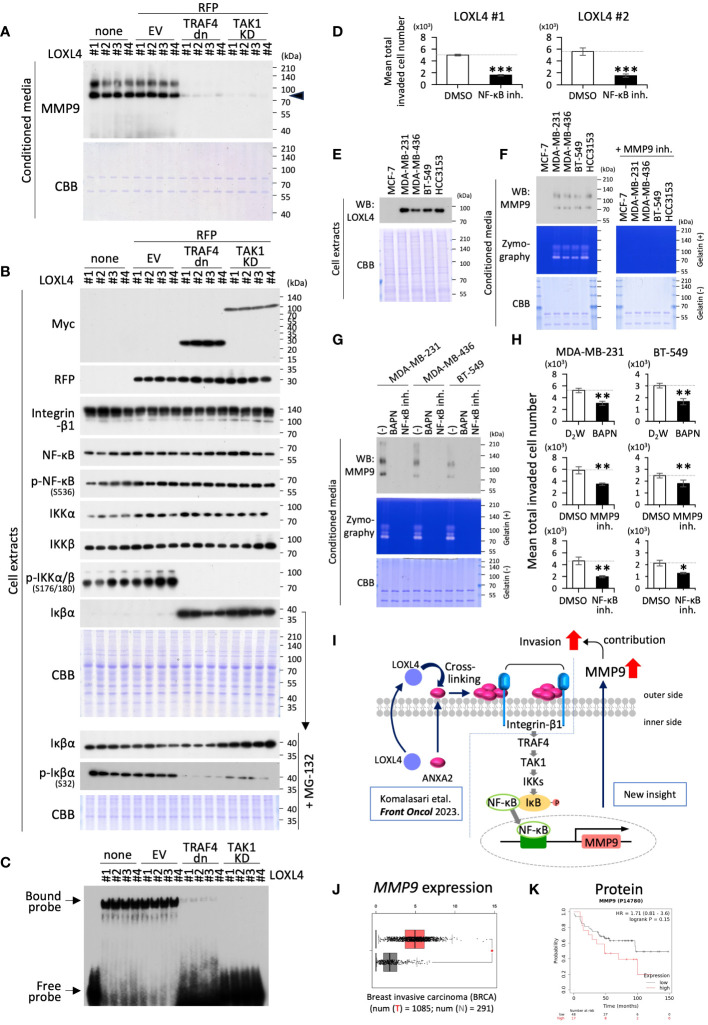
The beneficial role of TRAF4 and TAK1 in regulating MMP9 via NF-κB activation. **(A, B)** The indicated cells with transient transfection of the combination of RFP with the indicated genes (control empty vector EV, dominant negative TRAF4: TRAF4 dn, catalytically unfunctional kinase-dead TAK1: TAK1 KD) were collected by RFP-based cell sorting. None stands for intact non-treated cells with no cell sorting procedure, which were also used as control cells. WB analysis was done for the 10-fold condensed cell conditioned media and whole cell extracts from the LOXL4 wt-overexpressing clones to detect MMP9 **(A)** and the indicated protein molecules **(B)** following a procedure similar to that described in **Figures 2A, B**. **(C)** EMSA analysis of NF-κB was also performed for the whole extracts from the same series of cells, as shown in **(B)**, because there were too few cells to perform fractionation. **(D)** Invasion assay set with or without NF-κB inhibitor was carried out to evaluate the effect of NF-κB on the LOXL4-enhanced invasiveness. **(E, F)** Endogenous LOXL4 in cells and the secreted MMP9 in the conditioned media in the indicated cell lines were detected using the WB procedure **(E)**. On the other hand, zymography with gelatin substrate was performed to detect secretory MMP activity from the indicated cells. Specification of the MMP9-mediated digestive bands was done by the treatment of the gels with an MMP9-specific inhibitor **(F)**. Other running gels with no gelatin, also loaded with identical specimens corresponding to WB and zymography, were stained with CBB as sample controls of proper loading **(E, F)**. **(G)** The conditioned media prepared from the indicated cells treated or not treated with LOX inhibitor BAPN, MMP inhibitor, or NF-κB inhibitor were subjected to WB (upper) and zymography (lower) to detect their MMP9 proteins and activities. **(H)** A trans-chamber-based cell invasion assay also evaluated the effects of these inhibitors used in **(G)** on the invasive activities in the indicated cells. **(I)** Schematic diagram of the molecular interplay among the indicated vital molecules. **(J)** Gene expression plots of MMP9 mRNA from breast invasive carcinoma (BRCA) specimens were obtained from a publicly available website (http://gepia.cancer-pku.cn/). **(K)** Overall survival plots for MMP9 protein expression levels were obtained from a publicly available website (http://kmplot.com/analysis/). Data are mean ± SD. *p<0.05, **p<0.01, ***p<0.001.

The condensed cell-conditioned media prepared from the collected cell culture were first examined for MMP9 levels. We found that MMP9 induction was dramatically reduced by either TRAF4 dn- or TAK1 KD-overexpression in the clones that constantly overexpressed the LOXL4 wt (**Figure 4A**). As a result, WB analysis for the cellular proteins of interest further showed that phosphorylation of IKKα/β was almost diminished, and conversely, the Iκβα protein levels were highly increased (**Figure 4B**). We confirmed that the phosphorylation of Iκβα was significantly reduced in the presence of MG-132. Further, these results mirrored the EMSA results; that is, both sets of results indicated that NF-κB activation was greatly impeded by the forced induction of either TRAF4 dn- or TAK1 KD in the LOXL4 wt-overexpressing clones (**Figure 4C**). In line with the result that the MMP9 inhibition worked to suppress the LOXL4-mediated invasive activity of the LOXL4 clones, NF-κB inhibition should be similar to that with the MMP9 inhibition (**Figure 1E**). Within the expectation, we found that the active invasive motility of the LOXL4 clones was pronouncedly stalled by the treatment with NF-κB inhibitor (**Figure 4D**). We then examined the avid contribution of the vital molecules LOXL4, NF-κB, and MMP9 to the invasive activity of the non-gene-engineered TNBC cell lines. As shown in **Figure 4E**, the indicated TNBC cell lines used exhibited high expression of LOXL4 consistently as compared with that in the non-TNBC MCF-7 cells, which was also similarly observed in our previous report (10). Their elevated events of the intrinsic LOXL4 were matched with the high protein contents (**Figure 4E**) and enzymatic activities (**Figure 4F**) of MMP9 in their conditioned media. Of our interest, the inhibition approach of each LOXL4 and NF-κB commonly dampened the MMP9 at protein appearance and enzymatic gelatin digestive activity in the conditioned media prepared from the indicated three TNBC cell lines (**Figure 4G**). In concurrent with this, we found that the invasive activities of the indicated two TNBC cells were significantly lowered by every treatment with LOX inhibitor (BAPN), MMP9 specific inhibitor, or NF-κB inhibitor, consistently (**Figure 4H**). Taken together, our results provide new insight into the signal transduction of the LOXL4-mediated invasion—namely, the invasion involves the TRAF4-TAK1-IKKα/β-Iκβα-NF-κB-MMP9 axis (**Figure 4I**). Finally, our in-silico analysis based on a public database revealed significantly elevated expression of MMP9 in the invasive breast cancer tissues (**Figure 4J**) and poor survival in breast cancer patients with high expression of MMP9 (**Figure 4K**).

## 4 Discussion

In this study, we report for the first time the mechanism tethering LOXL4 and MMP9 in TNBC cells. Our previous study showed that LOXL4 exhibits the highest expression among the LOX family in TNBC cells. LOXL4 plays a crucial role in the invasive outgrowth of TNBC cells *in vitro* and *in vivo* (10). In addition to the well-known classical function of LOXL4—that is, collagen cross-linking that gives collagen its preferential stiffness, fashioning the collagen into an optimal component of a cancer-progressive milieu—we also succeeded in identifying another vital substrate on the TNBC cell surface, annexin A2. The LOXL4-mediated cross-linked annexin A2 suppresses the integrin-β1 uptake, whereby its enrichment on the cell surface functions in cancer-invasive outgrowth. However, how the integrin-β1-mediated signal(s) runs was not previously elucidated. In this study we uncovered a signal pathway that is linked to the TNBC cell invasiveness via the following molecular flow: integrin-β1-TRAF4-TAK1-IKKα/β-Iκβα-NF-κB-MMP9.

It is evident that MMP9 plays a pivotal role in the invasive outgrowth of TNBCs, as seen in our in-silico analysis (**Figures 4J, K**) and cell-based assay (**Figure 1E**). However, in the notion of **Figure 1E**, MMP9 looks similar to being involved in part of the LOXL4-mediated upregulation of invasiveness since the suppression rate is weak, not archive to decrease in the levels of control GFP cells as shown in **Figure 1A**, implying additional involvement of still unidentified mechanisms brought by LOXL4. Cell surface annexin A2 may bind a molecule other than integrin-β1 to contribute to the enhanced invasiveness. Annexin A2 on the cell surface can form a complex with S100A10 that functions as a substrate to capture plasminogen and its activating enzymes such as tissue-type plasminogen activator (tPA) or urokinase-type plasminogen activator (uPA), both of which catalyze the conversion of plasminogen to plasmin, so the plasmin on annexin A2/S100A10 facilitates invasive dissemination in cancer cells, since plasmin functions as a digestive enzyme of not only fibrin but also collagen as substrates in the manner of MMPs (17–20). We are interested in cell surface plasmin, which induces the enzymatic maturation of MMP9 (21–23). In addition, plasmin induces a functional active-type of TGF-β from the masked inactive-type of TGF-β, termed latent TGF-β, by digesting it (24). The ripe TGF-β is a central factor in the epithelial-mesenchymal transition (EMT) that is closely associated with cancer invasive dissemination (24, 25). Thus, the subjects described above are our ongoing areas of interest.

The interactive recruitment of TRAF4, followed by TAK1, to the integrin-β1 had not yet been reported when we conducted this study. This new finding is beneficial to disentangle the signal regulatory maze that tethers the signal space between integrin-β1 and IKKα/β-mediated activation of NF-κB. The significance of TRAF4/TAK1 interaction in the invasion-associated NF-κB activation is reinforced in the review report by Rousseau et al. (26), where they argue that the membrane local architecture tight junctions (TJs) allow fetching of the complex of TRAF4 and TAK1, eventually leading to NF-κB activation and an accelerating of invasion of cancer cells. Zhang et al. (27) have also demonstrated that TRAF4 is required for efficient cancer cell events such as EMT, migration, and metastasis in breast cancer cells via activation of TAK1 upon TGF-β stimulation. In addition, TAK1 has been shown to activate NF-κB through the phosphorylation of IKKα/β and promotes metastasis of TNBC cells (28, 29). These facts support our identified pathway in TNBC cells for their invasive activation triggered by LOXL4.

Another aspect that should be discussed is the potential force of MMP9 induction by a LOX family member other than LOXL4, since all LOX family members have an intrinsic catalytic domain that is responsible for the post-translational oxidative deamination of peptidyl lysine residues in collagen precursor (30). We established another clone that overexpresses individual family members, LOX wt, LOXL1 wt, LOXL2 wt, and LOXL3 wt, other than LOXL4 wt (**Supplementary Figure 2B**). The positive clones for the foreign genes transduced were chosen in an unbiased manner, and the cell-conditioned media were collected and subjected to zymography. As a result, we found that not only LOXL4 but also LOXL3 has the potential to induce MMP9 (**Supplementary Figure 2C**). This result was in good agreement with our previous finding that LOXL3 wt-overexpressing clone #1 acquired very active invasiveness like that by LOXL4 wt-overexpression (9). LOXL3 expression was not dominant compared to LOXL4 in TNBC cells, so LOXL3-mediated MMP9 functions in cancers other than TNBC. The MMP9 induction mechanism by LOXL3 will be altered from that of LOXL4 since LOXL4 cannot bind cell surface annexin A2 as a substrate. However, as to the reason that the canonical pathway leading to NF-kB activation is regulated by several membrane proteins (**Figure 3A**), we speculate that LOXL3 may induce MMP9 via as-yet-unidentified canonical pathway-relevant membrane proteins.

We mentioned the unusual role of LOXL4 rather than other LOX family members in the TNBC progression standing on the side of our new aspect of LOXL4; however, owing to the pleiotropic role of LOX family members including a matrices’ cross-linking function. All LOX family members may be vital in the TNBC when linked with their mal-overexpression of any one among them. Thus, LOX family members will become potent druggable targets for TNBC. LOX family-targeted therapies are therefore being developed to attack cancer cells and the cancer stroma. A lipid-based nanoparticle chemically conjugated with an inhibitor of LOX and loaded with epirubicin demonstrated significant efficacy in inhibiting cancer growth in an orthotropic xenograft mouse model of human TNBC (31). Additionally, LOX, which is induced by hypoxia-inducible factor 1 alpha (HIF-1α) in hypoxic environments, has been identified as a crucial factor in the development of chemotherapy resistance in TNBC. The overexpression of the LOX/ITGA5/FN1 axis has significantly reduced the survival of TNBC patients undergoing chemotherapy (32). The LOX family is confirmed to be highly expressed in both primary and metastatic lesions of most solid tumors. This suggests the potential benefits of combination therapy with LOX inhibitors and chemotherapy in various cancers and indicates the possibility of its use as a companion diagnostic (33).

In conclusion, we revealed a part of a new pathway of LOXL4, integrin-β1-TRAF4-TAK1-IKKα/β-Iκβα-NF-κB-MMP9, that plays a crucial role in TNBC cell invasiveness. This finding may contribute to the generation of LOXL4-signal cascade-targeted therapy to treat metastatic TNBC effectively. In support of this idea, Gorogh et al. (34) have reported that LOXL4 monoclonal antibody (LOXL4-mAb) effectively prevents head and neck squamous cell carcinoma (HNSCC) cells *in vivo*. Hence, in addition to the ongoing studies of the LOX-targeted cancer therapies as described above, we conjecture that the LOXL4 antibody or our pathway inhibitory chemicals, if designed, may also shed light on treating TNBC, which is very difficult to cure.

There are some limitations to our investigation about the clinical relevance of the identified pathway and its function in the mouse model in detail, so the theme is in our future direction.

## Data availability statement

The raw data supporting the conclusions of this article will be made available by the authors, without undue reservation.

## Ethics statement

Ethical approval was not required for the studies on humans in accordance with the local legislation and institutional requirements because only commercially available established cell lines were used.

## Author contributions

FJ: Methodology, Data curation, Writing – review & editing, Writing – original draft, Investigation, Formal analysis. YC: Methodology, Formal analysis, Data curation, Writing – review & editing, Investigation, Funding acquisition. NT: Writing – review & editing, Investigation, Funding acquisition. RK: Writing – review & editing, Investigation, Funding acquisition. NK: Writing – review & editing, Investigation. CK: Writing – review & editing, Investigation. KN: Writing – review & editing, Investigation. HM: Writing – review & editing, Investigation. KY: Writing – review & editing, Investigation. YG: Writing – review & editing, Investigation. TO: Writing – review & editing, Investigation. IR: Writing – review & editing, Investigation. IS: Writing – review & editing, Investigation. JZ: Writing – review & editing, Investigation. TH: Writing – review & editing, Investigation. YS: Writing – review & editing, Methodology, Investigation. AY: Writing – review & editing, Formal analysis, Data curation. FK: Writing – review & editing, Formal analysis, Data curation. JF: Writing – review & editing, Formal analysis, Data curation. EK: Writing – review & editing, Formal analysis, Data curation. YI: Writing – review & editing, Investigation. ST: Writing – review & editing, Formal analysis, Data curation. MS: Supervision, Project administration, Investigation, Funding acquisition, Conceptualization, Writing – review & editing, Writing – original draft.
