# Supplementary material for: Dissection of the signal transduction machinery responsible for the lysyl oxidase-like 4-mediated increase in invasive motility in triple-negative breast cancer cells: mechanistic insight into the integrin-β1-NF-κB-MMP9 axis
Source: Front Oncol. 2024 May 28;14:1371307. doi: 10.3389/fonc.2024.1371307 (PMC11165029; doi:10.3389/fonc.2024.1371307)
Supplement: Supplementary file 1 [file DataSheet_1.docx]

Supplementary Material

Dissection of the signal transduction machinery responsible for the lysyl oxidase-like 4-mediated increase in invasive motility in triple-negative breast cancer cells: mechanistic insight into the integrin-β1-NF-κB-MMP9 axis

Fan Jiang^1†^, Youyi Chen^2†^, Nahoko Tomonobu^1^, Rie Kinoshita^1^, Ni Luh Gede Yoni Komalasari^3^, Carlos Ichiro Kasano-Camones^4^, Kazumi Ninomiya^4^, Hitoshi Murata^1^, Ken-ichi Yamamoto^1^, Yuma Gohara^1^, Toshiki Ochi^1,5^, I Made Winarsa Ruma^3^, I Wayan Sumardika^3^, Jin Zhou^6^, Tomoko Honjo^7^, Yoshihiko Sakaguchi^8^, Akira Yamauchi^9^, Futoshi Kuribayashi^9^, Junichiro Futami^7^, Eisaku Kondo^10^, Yusuke Inoue^4^, Shinichi Toyooka^11^, Masakiyo Sakaguchi^1*^

^1^ Department of Cell Biology, Okayama University Graduate School of Medicine, Dentistry and Pharmaceutical Sciences, Okayama, Japan.

^2^ Department of Breast Surgery, The First Affiliated Hospital, Zhejiang University School of Medicine, Hangzhou 310003, PR. China.

^3^ Faculty of Medicine, Udayana University, Denpasar, Bali, Indonesia.

^4^ Faculty of Science and Technology, Division of Molecular Science, Gunma University, Kiryu, Gunma, Japan.

^5^ Department of Neurology, Okayama University Graduate School of Medicine, Dentistry and Pharmaceutical Sciences, Okayama, Japan.

^6^ Medical Oncology Department of Gastrointestinal Tumors, Liaoning Cancer Hospital & Institute, Cancer Hospital of the Dalian University of Technology, Shenyang, Liaoning, China.

^7^ Department of Interdisciplinary Science and Engineering in Health Systems, Okayama University, Okayama, Japan.

^8^ Department of Microbiology, Tokushima Bunri University, Sagamihara, Tokushima, Japan.

^9^ Department of Biochemistry, Kawasaki Medical School, Kurashiki, Okayama, Japan.

^10^ Division of Tumor Pathology, Near InfraRed Photo-Immuno-Therapy Research Institute, Kansai Medical University, Osaka, Japan.

^11^ Department of General Thoracic Surgery and Breast and Endocrinological Surgery, Okayama University Graduate School of Medicine, Dentistry and Pharmaceutical Sciences, Okayama, Japan.

^†^These authors contributed equally to this work and share the first authorship.

***Corresponding Author**: Masakiyo Sakaguchi, Ph.D.
E-mail: [masa-s@md.okayama-u.ac.jp](mailto:masa-s@md.okayama-u.ac.jp); Phone number: +81-86-235-7395; Fax: +81-86-235-7400; Department of Cell Biology, Okayama University Graduate School of Medicine, Dentistry and Pharmaceutical Sciences, 2-5-1 Shikata-cho, Kita-ku, Okayama-shi, Okayama 700-8558, Japan

# Supplementary Figures

## Supplementary Figure 1.

**SUPPLEMENTARY FIGURE 1. Establishment of the genetically engineered sublines that express control GFP or LOXL4 wt based on MDA-MB-231 cells as parental cells and reporter assays of NF-κB-mediated MMP9 promoter activation. A,** Representative images of immunofluorescence staining of LOXL4 in the breast cancer tissues (stage III) were displayed. Nuclei were stained with SYBR Green. **B,** Establishment of MDA-MB-231-derived sublines that stably express foreign GFP or LOXL4 wt. The LOXL4 was designed to have an HA tag on the C-terminal side. The stable clones numbered in red were chosen in an unbiased manner. **C, D,** A real-time qPCR was performed to determine the endogenous levels of MMP family mRNAs in the indicated cells. TBP mRNA was used as a control for the analysis. Total RNA was extracted from the cultured cells using an RNeasy Mini Kit (Qiagen, Venlo, Netherlands). Reverse transcription was then performed using ReverTraAce qPCR RT Master Mix with gDNA Remover (Toyobo, Osaka, Japan). A real-time polymerase chain reaction (PCR) assay was performed on a LightCycler 480 system II (Roche Applied Science, Penzberg, Germany) for all gene transcripts of interest using FastStart SYBR® Green Master Mix (Roche Applied Science) with specific primers (**C**). The expression levels of MMPs were normalized relative to TBP mRNA as an internal control using the ΔΔCt method, repeated three times for each set of samples, and summarized as bar graphs (**D**). **E, F,** NF-κB-mediated promoter activation of MMP9 was evaluated by luciferase-based reporter gene assay using the NF-κB-reporter plasmid (**E**) and MMP9 promoter reporter plasmid (**F**). The -3982/+146 fragment from the transcription start site of the human MMP9 promoter was amplified with genomic DNA from Huh7 cells by PCR and cloned into the luciferase reporter vector pGL4.11 (Promega) (**F**). The primers used to amplify the promoter were 5’-GAGCTCTCCCCTGAGGCAATGTCTTG-3’ and 5’-CTCGAGGCCTGTCGGTGAGATTGGTT-3’. pGL4.11 carrying the human MMP9 promoter and pGL4.74 encoding Renilla luciferase regulated under the HSV-TK promoter as an internal control were transfected into HEK293T cells with polyethyleneimine Max (Polyscience, Warrington, PA) as a transfection reagent. For co-transfection, pCMViR-TSC vectors carrying LOXL4 expression plasmids (wt and mutCA) were used to monitor the MMP9 promoter. After 48 h, transfected cells were measured using a Dual-Glo Luciferase Assay System (Promega BioSciences). In a similar manner, pNF-κB-Luc plasmid (Takara Bio USA) was used to assess LOXL4-mediated NF-κB activation (**E**). HEK293T cells were co-transfected with pNF-κB-Luc or pGL4.14-pMMP9-Luc and LOXL4 wt expression plasmid, and the level of expressed luc activities was measured. Data are mean ± SD. ND: not detected, *p<0.05, **p<0.01, ***p<0.001.

## Supplementary Figure 2.

**SUPPLEMENTARY FIGURE 2. Evaluation of the delivered foreign genes, TRAF4 dn or TAK1 KD with GFP, double-positive cells in MDA-MB-231 cells and evaluation of MMP9 production of the genetically engineered sublines that express all LOX family members based on MDA-MB-231 cells as parental cells. A,** Double immunofluorescence staining of either TRAF4 dn (red color) or TAK1 KD (red color) and GFP (green color) was performed in MDA-MB-231 cells. The top panel shows a double staining image of TRAF4 dn and GFP at magnification x10. The lower panels are enlargements of the staining of TRAF4 dn and GFP or TAK1 KD and GFP at magnification x40. **B,** Establishment of MDA-MB-231-derived sublines that stably express foreign LOX, LOXL1, LOXL2, or LOXL3, designed to have an HA tag at the C-terminal side. **C,** The 10-fold condensed cell-conditioned media from the indicated cell cultures were subjected to gelatin zymography. The arrowhead shows the digested bands by MMP9.

**
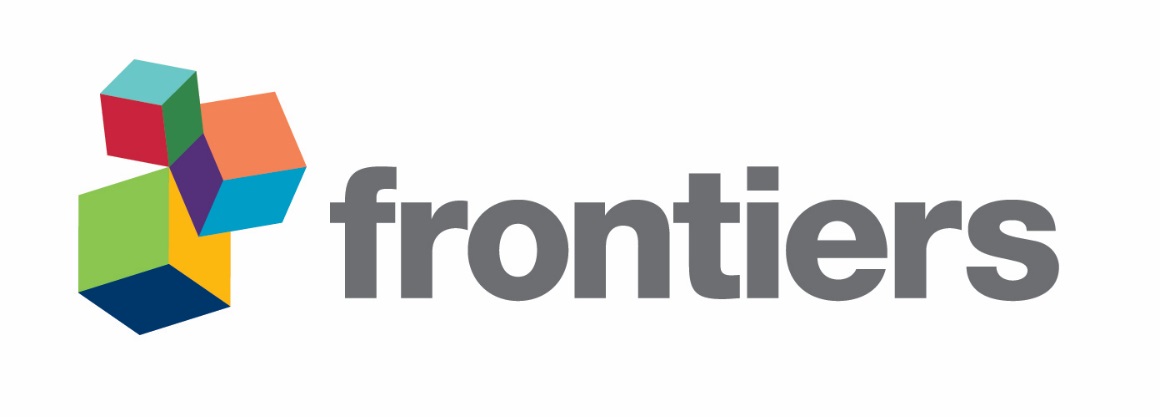
**
